# When strokes reveal a hidden malignancy: An atypical case of metastatic colorectal cancer with extensive thromboembolism

**DOI:** 10.18632/oncoscience.626

**Published:** 2025-09-15

**Authors:** Md Tanzim Ahsan, Sultana Azreen

**Affiliations:** ^1^Wrightington, Wigan and Leigh (WWL) Teaching Hospitals, NHS Foundation Trust, The United Kingdom; ^2^Clatterbridge Cancer Centre, Clatterbridge Rd, Birkenhead, Wirral, Merseyside, The United Kingdom

**Keywords:** trousseau’s syndrome, cancer-associated stroke (CAS), hypercoagulability, colorectal cancer, systemic thromboembolism

## Abstract

Cancer-associated thromboembolism and Trousseau’s syndrome, which are paraneoplastic hypercoagulable states, are significant yet frequently underrecognized causes of cryptogenic stroke. We report a 71-year-old male who presented with recurrent embolic strokes and systemic thrombosis, later diagnosed with metastatic colorectal adenocarcinoma. Despite extensive investigations, no initial cancer-related symptoms were noted, and the diagnosis was made retrospectively after supraclavicular lymph node biopsy. The patient exhibited multi-territory infarcts, deep vein thrombosis, and renal and splenic infarcts, highlighting the aggressive thrombotic potential of malignancy-associated stroke. This case highlights the importance of early consideration of occult malignancy in cryptogenic embolic events, supporting comprehensive oncologic evaluation for unexplained hypercoagulable states.

## INTRODUCTION

Cancer poses a considerable burden to global health, with an estimated lifetime risk of 40% and a reported approximately 20 million new cancer cases and 9.7 million cancer-related deaths worldwide in 2022 [1, 2]. Aside from its direct impact, malignancy is responsible for systemic sequelae, one of which is a heightened risk of thromboembolism, which presents as ischemic stroke [3]. Cancer-related thromboembolism, including Trousseau’s Syndrome and Cancer-Associated Stroke (CAS), is becoming increasingly recognized as a significant clinical concern. Research indicates that nearly 10% of ischemic stroke cases may be linked to an underlying cancer [4]. The risk of ischemic stroke is significantly elevated in cancer patients, particularly in the months preceding or following a cancer diagnosis, indicating a potential link between cryptogenic strokes and occult malignancy [5].

Cancer-associated thrombosis is caused by several mechanisms, such as hypercoagulability, non-bacterial thrombotic endocarditis (NBTE), direct invasion of the vessel, and endothelial dysfunction due to chemotherapy [4]. Among these, NBTE poses a particularly alarming risk for ischemic stroke as it leads to embolization without any signs of a bacterial infection [3]. In a large autopsy study, cerebrovascular disease was detected in 14.6% of cancer patients, with nearly half of these strokes being clinically silent [5]. Additionally, systemic cancer enhances thrombin generation, promoting both venous and arterial thrombosis [4].

Systemic thrombosis, like multifocal arterial and venous embolism, also hinders early diagnosis, postponing the diagnosis of an underlying malignancy [6]. This is also compounded by the fact that cancer screening approaches in stroke are contentious, and there is no standard approach to finding occult malignancies in stroke patients [5]. For instance, colorectal cancer, despite being among the most common malignancies worldwide, is less frequently implicated in stroke-related thromboembolism compared to lung or pancreatic cancers [1, 7]. However, a recent study of over 7.5 million cancer patients found colon cancer to be among the leading malignancies to cause fatal strokes [1].

We present a unique case of metastatic colon adenocarcinoma that manifested with recurrent embolic strokes and significant thrombosis. This highlights the necessity of maintaining a high suspicion for cancer in patients experiencing cryptogenic thromboembolic events. By sharing this case, we aim to enhance the existing literature on cancer-induced strokes and emphasize the necessity for improved screening guidelines for individuals presenting with cryptogenic strokes and systemic thrombosis.

## CASE PRESENTATION

A 71-year-old male was admitted to the hospital on December 31, 2024, with a three-week history of generalized malaise, left-sided headache, and swelling of the left upper limb. Although symptoms raised initial suspicion for vasculitis or autoimmune etiologies due to limb swelling and headache, he did not report associated visual disturbances, nausea, vomiting, or neurological deficits. He denied experiencing pain while chewing or touching his face and did not have any symptoms suggestive of temporal arteritis. There were no night sweats, significant weight loss, or symptoms indicative of systemic infection, although he had been experiencing intermittent hot flushes. His only gastrointestinal symptom was occasional constipation, but he denied diarrhea, abdominal pain, or urinary complaints.

His past medical history was significant for hypertension, type 2 diabetes mellitus, ulcerative colitis, and osteoarthritis. His regular medications included atorvastatin 40 mg once daily, amlodipine 10 mg once daily, indapamide 2.5 mg once daily, sulfasalazine 500 mg twice daily, ramipril 10 mg once daily, and metformin 500 mg modified-release twice daily. He lived with his wife, was a non-smoker, and consumed alcohol occasionally. He had been independently mobile with the use of a walking stick but had noted a recent decline in mobility due to worsening knee pain.

On examination, his vital signs revealed a blood pressure of 175/75 mmHg, heart rate of 88 beats per minute, respiratory rate of 20 breaths per minute, and oxygen saturation of 97% on room air. He was afebrile at the time of presentation, with a recorded temperature of 37.6°C. He was alert and oriented, and there were no signs of acute distress. Cardiovascular examination was unremarkable, and auscultation of the chest revealed good bilateral air entry with no evidence of consolidation. Examination of the head and neck identified right submandibular lymph node enlargement, which was non-tender and non-matted, as well as a non-pulsatile, non-tender left supraclavicular swelling, raising suspicion for lymphadenopathy. There were no palpable axillary lymph nodes. A notable generalized swelling of the left upper limb was present, with preserved power and sensation. Additionally, a cystic swelling was observed over the left elbow. There were no oral ulcers or dental abscesses, and throat examination was unremarkable. On inspection of the chest, telangiectasia was noted over the left pectoral region. His abdominal examination was normal, and he exhibited a wobbly gait.

### Investigations and initial workup

Initial blood work demonstrated a markedly elevated C-reactive protein (CRP) at 119 mg/L, along with leukocytosis (WBC 15 × 10^9^/L, with neutrophils 12.3 × 10^9^/L). Hemoglobin was 110 g/L, and platelets were within normal limits at 161 × 10^9^/L. His kidney function was preserved, with an estimated glomerular filtration rate (eGFR) above 90 mL/min/1.73 m², although his serum potassium was low at 2.8 mmol/L. His random blood glucose was elevated at 9.7 mmol/L.

**Figure 1:**
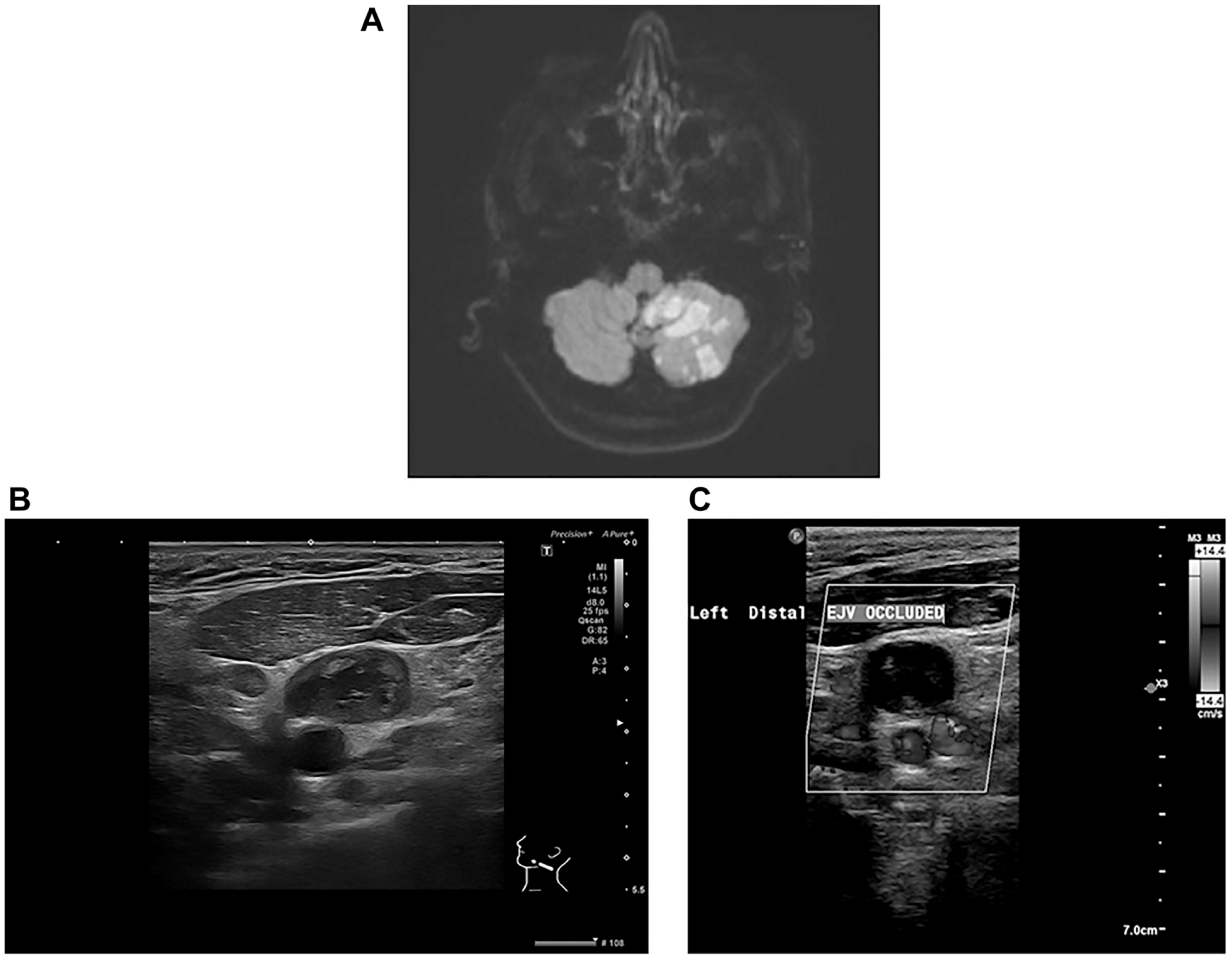
Multi-modality imaging findings in a patient with Trousseau’s syndrome and metastatic colorectal cancer. (**A**) Diffusion-weighted MRI of the brain demonstrating multifocal acute infarcts in multiple vascular territories, consistent with an embolic event. (**B**) Ultrasound of the neck revealing an enlarged supraclavicular lymph node with internal heterogeneity, raising suspicion for metastatic involvement. (**C**) Doppler ultrasound of the left neck demonstrating complete occlusion of the external jugular vein (EJV), consistent with malignancy-associated thrombosis.

A chest X-ray showed no evidence of consolidation. Electrocardiography (ECG) revealed normal sinus rhythm, and a focused assessment with sonography for trauma (FAST) was negative. Given his neurological symptoms, an MRI of the brain was performed, which demonstrated multifocal acute infarctions in multiple vascular territories, most consistent with a cardioembolic event (Figure 1A).

### Progression and further workup

Following admission, his clinical course raised concerns regarding an underlying prothrombotic state. A computed tomography (CT) scan of the neck, chest, abdomen, and pelvis performed on December 31, 2024, revealed multiple enlarged left supraclavicular and cervical lymph nodes, extensive intra-abdominal para-aortic lymphadenopathy, and several nodular lesions in the anterior pararenal space, raising suspicion for a malignant lymphoproliferative disorder (Figure 1B). In addition, extensive thrombosis was noted in the left internal jugular vein and subclavian vein. There was also evidence of wedge-shaped bilateral hypodense cortical areas in the middle and lower thirds of both kidneys, suggestive of renal infarcts.

Further Doppler ultrasonography on January 2, 2025, confirmed extensive deep vein thrombosis (DVT) in the left upper limb (Figure 1C). A repeat CT scan on January 9, 2025, showed significant progression of the mesenteric and para-aortic lymphadenopathy, reinforcing concerns for an underlying malignancy, particularly lymphoma. Additional findings included a newly identified splenic infarct and persistent renal infarcts. Pulmonary emboli were also noted, though of small volume.

Given the constellation of embolic events, the hematology team initiated therapeutic anticoagulation with Dalteparin. Cardiology was consulted, and an echocardiogram performed on December 30, 2024, ruled out infective endocarditis, showing no valvular vegetations. A serial troponin trend revealed an initial elevation (1354 ng/L), which subsequently declined (1206 ng/L to 974 ng/L), making an acute coronary syndrome less likely. Telemetry monitoring did not detect any episodes of atrial fibrillation.

The patient’s condition continued to deteriorate, prompting a biopsy of the left supraclavicular lymph node on January 17, 2025. Histopathological analysis revealed metastatic adenocarcinoma with immunohistochemical markers strongly positive for AE1/AE3, CK20, and CDX2, suggesting a colorectal primary. The malignant cells were negative for CK7, TTF-1, Napsin A, GATA 3, and PAX 8. Based on these findings, further investigation into a primary colorectal malignancy was recommended.

### Hospital course and outcome

Despite medical management, the patient continued to decline, with progressive lymphadenopathy and worsening systemic symptoms. His clinical condition raised concerns about a new cerebrovascular event, prompting an urgent CT of the head on January 10, 2025, which confirmed an acute left parietal infarct, along with subacute infarctions in the left cerebellar hemisphere and occipital lobe. Given his multiple embolic episodes and high suspicion of an underlying malignancy, he remained on anticoagulation, which was transitioned from dalteparin to apixaban 5 mg twice daily at discharge (see Table 1 for an overview of clinical findings).

**Table 1:** Overview of key clinical findings in the case

Category	Findings	Timeline (Date)
Initial Presentation	Generalized malaise, left-sided headache, left upper limb swelling, occasional constipation, intermittent hot flushes.	December 31, 2024
Neurological Events	Multifocal acute infarcts in various vascular territories (left parietal, cerebellar, occipital), unsteady gait.	MRI on admission (December 31, 2024), repeat CT on January 10, 2025
Cardiovascular	Normal sinus rhythm on ECG, transient elevation of troponin levels, no vegetations on echocardiogram.	ECG and Echocardiogram (December 30-31, 2024)
Respiratory	Small-volume bilateral pulmonary embolism	CT (January 9, 2025)
Lymphatic System	Progressive supraclavicular, cervical, para-aortic lymphadenopathy; biopsy confirming metastatic colorectal adenocarcinoma.	Biopsy (January 17, 2025)
Hematologic Parameters	Leukocytosis (WBC: 15 × 10^9^/L, neutrophils: 12.3 × 10^9^/L), Hemoglobin: 110 g/L, platelets normal, elevated CRP (119 mg/L, subsequently reducing to 57 mg/L).	December 31, 2024 – January 2025
Thrombotic Complications	Extensive thrombosis (left internal jugular vein, subclavian vein, brachiocephalic veins), renal and splenic infarcts, confirmed extensive left upper limb deep vein thrombosis.	Doppler Ultrasound (January 2, 2025), CT scans (December 31, 2024 and January 9, 2025)
Malignancy Workup	Metastatic adenocarcinoma confirmed with positive CK20, CDX2, negative CK7, TTF-1, suggesting colorectal origin.	Immunohistochemistry (January 17, 2025)

Following discussions with the stroke team, the hematology service, and his family, a decision was made to focus on palliative care. Given his rapid clinical deterioration and poor performance status, chemotherapy was considered but ultimately ruled out by multidisciplinary consensus, and he was subsequently discharged under a Fast Track discharge pathway to a nursing home. A multidisciplinary team (MDT) discussion on February 3, 2025, confirmed that he was not a candidate for further intervention due to his advanced disease. He remained under supportive care.

## DISCUSSION

This case report describes a 71-year-old male who presented with recurrent embolic strokes and systemic thrombosis, ultimately diagnosed with metastatic colorectal adenocarcinoma. Despite lacking overt cancer symptoms, the patient experienced multiple embolic strokes and extensive systemic thrombosis, eventually diagnosed retrospectively with metastatic colorectal adenocarcinoma. This case highlights diagnostic challenges and emphasizes inflammation-driven malignancy transformation, underscoring the importance of early suspicion for occult malignancy in cryptogenic thromboembolism. The case illustrates the importance of maintaining a high index of suspicion for occult cancer in patients presenting with unexplained stroke and systemic thrombosis. Earlier suspicion or targeted screening could have potentially enabled timely initiation of oncologic therapy, possibly influencing clinical outcomes.

Trousseau’s syndrome (TS), a paraneoplastic hypercoagulability syndrome, is considered a subset of CAS, primarily manifesting as arterial and venous thromboembolism in cancer patients. The relationship between TS and CAS is critical, as both conditions share common coagulation pathway activations driven by tumor-secreted factors [8]. Kitamura et al. described TS in hematologic malignancies, where embolic strokes resulted from hypercoagulability linked to cytokine-driven endothelial activation [8]. Similarly, Chen et al. presented cases where systemic thrombosis preceded cancer diagnosis, reinforcing the importance of comprehensive oncologic screening in embolic stroke patients without clear etiologies [9] (Table 2).

**Table 2:** Comparative overview of published trousseau’s syndrome cases

Study/Year	Cancer type	Case	Presentation	D-Dimer	Treatment	Outcome
Liu et al., 2023 [6]	Various malignancies	Female, 54 yrs	Cerebral infarcts, myocardial injury, renal infarct, DVT	Elevated	Aspirin, LMWH	Fatal
Wakabayashi et al., 2023 [16]	Pancreatic cancer	Various patients	Systemic embolism	Elevated	Chemotherapy, anticoagulation	Poor prognosis
Tasi et al., 2004 [14]	Colorectal, cholangiocarcinoma	Females, 43 and 57 yrs	Refractory thromboembolism	Elevated	LMWH	Fatal
Chen et al., 2024 [9]	Cervical, gastric cancer	Females, 67 and 48 yrs	Stroke-like symptoms, deep vein thrombosis	Elevated	LMWH	Clinical improvement
Meng et al., 2024 [13]	Gastrointestinal malignancy	Female, 69 yrs	Recurrent cerebral infarction	Elevated	Aspirin, statins, anticoagulants	Fatal within 1 year
Morales Eslava et al., 2024 [7]	Non-Hodgkin’s lymphoma	Female, 72 yrs	Deep vein thrombosis, pulmonary embolism	Elevated	LMWH, warfarin	Fatal
Kitamura et al., 2024 [8]	Diffuse large B-cell lymphoma	Female, 62 yrs	Multiple cerebral infarcts	Elevated	Rituximab, chemotherapy	Clinical improvement
**Current Case,** 2025	Colorectal adenocarcinoma	Male, 71 yrs	Multifocal cerebral infarcts, systemic embolism (renal, splenic), extensive DVT and pulmonary embolism	Elevated	LMWH, transitioned to apixaban, supportive care	Palliative care (Fatal)

The mechanisms underlying CAS are largely driven by cancer-induced hypercoagulability, with malignancies promoting systemic thrombosis through increased thrombin generation, platelet activation, and procoagulant microparticles [10]. Elevated tissue factor (TF) expression in tumor cells accelerates clot formation, while neutrophil extracellular traps (NETs) further contribute by providing a scaffold for thrombus development. Currently, the measurement of TF and soluble P-selectin remains largely within research settings rather than routine clinical practice [10]. Key differences in biomarker profiles between Trousseau’s syndrome and non-cancer-associated thromboembolism are summarized in Table 3. Studies have demonstrated significantly higher NET levels in CAS patients, correlating with D-dimer elevations, which serve as potential biomarkers for detecting occult malignancies. Future research and clinical trials targeting NETs may offer promising avenues for therapeutic intervention in CAS [11]. In some cases, nonbacterial thrombotic endocarditis (NBTE) leads to sterile vegetations on cardiac valves, which embolize and cause multi-territory infarcts, as seen in our patient [12]. While tumor embolism is rare, it has been reported in colorectal cancer, further complicating stroke etiology [3, 10].

**Table 3:** Comparative biomarker profiles in trousseau’s syndrome vs. Non-cancer-associated thromboembolism

Biomarker	Trousseau’s syndrome	Non-cancer-associated thromboembolism	Clinical implication	References
D-dimer	Markedly elevated; often correlates with tumor burden and thrombotic activity.	Elevated, but typically lower than in cancer-associated cases.	High levels may indicate occult malignancy in unexplained thrombotic events.	[17]
Tissue Factor (TF)	Elevated due to tumor cell expression and release of TF-bearing microparticles.	Usually within normal range; not a primary driver of thrombosis.	Elevated TF suggests malignancy-driven coagulation activation.	[18, 19]
Fibrinogen	Elevated as part of acute-phase response and tumor-induced coagulation.	May be elevated in inflammatory states; less pronounced than in cancer cases.	Significant elevation supports a cancer-associated hypercoagulable state.	[20]
Soluble P-selectin	Increased due to platelet activation and endothelial interaction with tumor cells.	May be elevated in inflammatory conditions; levels generally lower than in cancer.	Elevated levels can aid in differentiating cancer-associated thrombosis.	[21, 22]
Prothrombin Fragment 1+2	Elevated, indicating increased thrombin generation linked to malignancy.	May be elevated in acute thrombosis; levels typically lower than in cancer cases.	High levels reflect enhanced coagulation activity, suggestive of cancer involvement.	[23, 24]
Thrombin-Antithrombin Complex (TAT)	Elevated due to continuous thrombin generation in cancer.	Elevated during acute events; returns to baseline post-resolution.	Persistent elevation may indicate ongoing malignancy-driven coagulation.	[25, 26]
Fibrin Degradation Products (FDPs)	Elevated, reflecting extensive fibrinolysis in response to widespread thrombosis.	Elevated in acute thrombosis; levels decrease with treatment.	Sustained elevation suggests persistent thrombotic activity, potentially due to cancer.	[27, 28]

Certain imaging and laboratory results differentiate CAS from other embolic strokes. Atrial fibrillation-free multi-territory infarcts are very suggestive of a hypercoagulable state [3]. Additionally, D-dimer levels are always elevated in CAS compared to the typical stroke mechanisms [11]. Management is contentious, with DOACs having proven to be of limited benefit in recurrent CAS prevention, and LMWH remaining the anticoagulant of choice for malignancy-related thrombosis [12]. With advanced illness in our patient, treatment prioritized supportive care over intense stroke prophylaxis.

The cases reported by Chen et al. and Meng et al. further demonstrated that cancer-associated embolic strokes often present with multiple infarcts in various vascular territories, reinforcing the importance of hypercoagulability assessment in cryptogenic strokes [10, 13]. Additionally, Tasi et al. and Liu et al. reported cases of gastrointestinal malignancies leading to refractory thromboembolism, similar to our patient’s presentation [14, 15]. These studies highlight the aggressive thrombotic potential of mucin-producing tumors and the need for early identification to guide therapeutic decisions (Table 2).

The importance of multimodal treatment strategies is underscored by Wakabayashi et al., who found that patients receiving concurrent cancer treatment and anticoagulation had significantly better outcomes than those managed with anticoagulation alone [16]. This suggests that treating the underlying malignancy is as crucial as anticoagulation in controlling CAS progression. Additionally, Tasi et al. highlighted the limited efficacy of standard anticoagulation in some cases, suggesting that adjunctive therapies targeting tumor-induced coagulation pathways may be beneficial [14] (Table 2).

Future research needs to maximize risk stratification using biomarkers like NETs and identify optimal anticoagulation therapy to improve CAS outcomes. Further investigations into the efficacy of alternative anticoagulants, such as novel direct thrombin inhibitors, may provide better long-term management strategies. Additionally, prospective studies assessing the utility of advanced imaging techniques, such as PET-CT and thrombus histopathology, could refine early cancer detection in patients presenting with embolic strokes of unknown origin. Ultimately, a multidisciplinary approach integrating neurology, oncology, and hematology expertise is essential to improving diagnostic accuracy and treatment efficacy for patients with CAS and TS.

## CONCLUSIONS

This case highlights the challenges of diagnosing Trousseau’s syndrome and the connection between idiopathic thromboembolism and malignancy, as well as the need for early screening for cancer in patients presenting with cryptogenic strokes. The patient’s recurring embolic events, deep vein thrombosis, and systemic infarctions were ultimately diagnostic of metastatic colorectal cancer, illustrating the challenge of diagnosing Trousseau’s syndrome. Although anticoagulation remains the cornerstone of therapy, recurrences are common, and more effective screening and treatment strategies are needed. Future research should be directed at the optimization of diagnostic approaches and anticoagulation regimes. Multidisciplinary approach is the cornerstone of the improvement of results in cancer-related stroke and hypercoagulability patients.
